# Loss of RDM1 enhances hepatocellular carcinoma progression via p53 and Ras/Raf/ERK pathways

**DOI:** 10.1002/1878-0261.12593

**Published:** 2019-12-19

**Authors:** Shi‐Lu Chen, Li‐Li Liu, Chun‐Hua Wang, Shi‐Xun Lu, Xia Yang, Yang‐Fan He, Chris Zhiyi Zhang, Jing‐Ping Yun

**Affiliations:** ^1^ State Key Laboratory of Oncology in South China Collaborative Innovation Center for Cancer Medicine Sun Yat‐sen University Cancer Center Guangzhou China; ^2^ Department of Pathology Sun Yat‐sen University Cancer Center Guangzhou China; ^3^ Key Laboratory of Functional Protein Research of Guangdong Higher Education Institutes Institute of Life and Health Engineering College of Life Science and Technology Jinan University Guangzhou China

**Keywords:** hepatocellular carcinoma, METTL3, p53, RDM1, RNA methylation

## Abstract

Hepatocellular carcinoma (HCC), with its ineffective therapeutic options and poor prognosis, represents a global threat. In the present study, we show that RAD52 motif 1 (RDM1), a key regulator of DNA double‐strand break repair and recombination, is downregulated in HCC tissues and suppresses tumor growth. In clinical HCC samples, low expression of RDM1 correlates with larger tumor size, poor tumor differentiation, and unfavorable survival. *In vitro* and *in vivo* data demonstrate that knockdown of RDM1 increases HCC cell proliferation, colony formation, and cell population at G2/M phase, whereas RDM1 overexpression results in the opposite phenotypes. Mechanistically, RDM1 binds to the tumor suppressor p53 and enhances its protein stability. In the presence of p53, RDM1 suppresses the phosphorylation of Raf and ERK. Overexpression of p53 or treatment with ERK inhibitor significantly abolishes cell proliferation induced by the depletion of RDM1. In addition, overexpression of methyltransferase‐like 3 markedly induces N6‐methyladenosine modification of RDM1 mRNA and represses its expression. Taken together, our study indicates that RDM1 functions as a tumor suppressor and may be a potential prognostic and therapeutic factor for HCC.

## Graphical abstract

Loss of RAD52 motif 1 (RDM1), induced by methyltransferase‐like 3 (METTL3)‐mediated m^6^A modification, correlates with unfavorable clinical outcomes, promotes hepatocellular carcinoma (HCC) cell growth, and induces G2/M cell cycle arrest. Overexpression of p53 or treatment with ERK inhibitor significantly abolishes cell proliferation induced by the depletion of RDM1. The newly identified METTL3/RDM1/p53/ERK axis provides potential prognostic and therapeutic targets for HCC treatment.

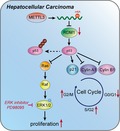

**Abbreviations**
5‐Aza‐CdR: 5‐aza‐2′‐deoxycytidine
CHX: cycloheximide
Co‐IP: immunoprecipitation
DMEM: Dulbecco’s modified Eagle’s medium
GSEA: Gene Set Enrichment Analysis
HCC: hepatocellular carcinoma
IF: immunofluorescence
IHC: immunohistochemistry
m^6^A: N6‐methyladenosine
METTL3: methyltransferase‐like 3
RDM1: RAD52 motif 1
TCGA: The Cancer Genome Atlas
TMA: tissue microarray

## 1 Introduction

Hepatocellular carcinoma (HCC) is the second leading cause of cancer death in men worldwide (El‐Serag, 2011; Villanueva and Llovet, 2014). Limited clinical treatments are available for HCC due to late‐stage diagnosis, fast tumor growth, and early postsurgery recurrence (Forner *et al.*, 2012). Dysregulation of cancer‐related genes is responsible for the carcinogenesis of HCC. Although a serious of oncogene and suppressor genes have been identified, the complex regulatory network of HCC remains largely unknown. Hence, elucidating the underlying molecular mechanisms of HCC progression is of great importance.

The RDM1 (RAD52 motif 1) gene is located on 17q11.2, encoding a 284‐amino‐acid protein. RDM1 is distributed in both nuclear and cytoplasmic compartments with diverse functions. The RDM1 protein contains two specific motifs, an RNA recognition motif (RRM) that is 90 aa and spans exon 1–2 and an RD motif that is 28 aa, spans exon 3, and is involved in DNA double‐strand break repair, RNA processing, and protein translation (Fan and Steitz, 1998; Horke *et al.*, 2004). Given the wide‐range biological roles of RDM1, we proposed that RDM1 might constitute an important aspect of cancer initiation and progression. Currently, there are few studies examining RDM1 in human cancers. In breast cancer, RDM1 is overexpressed compared to normal breast among DNA repair genes (Matta *et al.*, 2013). In lung adenocarcinoma, RDM1 is upregulated and correlates with poor clinical outcomes (Tong *et al.*, 2018). In papillary thyroid carcinoma, RDM1 plays an oncogenic role by inducing apoptosis and G2/M arrest (Li *et al.*, 2017a). However, the role of RDM1 in HCC has not been described.

Accumulating evidence of large‐scale sequencing proposed that epigenetic alterations are a hallmark of liver carcinogenesis (Coffelt and de Visser, 2014; Lee *et al.*, 2016). The physiological significance of RNA modification has been increasingly appreciated in recent years. Methyltransferase‐like 3 (METTL3) was originally identified as an oncogenic mRNA methylation (m^6^A, N6‐methyladenosine) catalyzing enzyme (Bokar *et al.*, 1997). The malignant role of METTL3 in promoting HCC growth and invasion suggests that METTL3 is a key factor for cell fate determination (Cai *et al.*, 2018; Chen *et al.*, 2018a; Cui *et al.*, 2017; Vu *et al.*, 2017). Identifying downstream effects of METTL3 may further elucidate its oncogenic properties.

In this study, we investigated the clinical significance and regulatory mechanisms of RDM1 in HCC. We demonstrate that RDM1 acts as a tumor suppressor in HCC by inhibiting cell proliferation. This newly identified METTL3/RDM1/p53/ERK axis supplements current understanding of HCC progression and provides potential prognostic and therapeutic targets for HCC treatment.

## 2 Materials and methods

### 2.1 Patients, tissue specimens, and follow‐up

A total number of 755 paraffin‐embedded primary HCC samples and corresponding nontumor tissues were obtained from Sun Yat‐sen University Cancer Center (SYSUCC). HCC patients who underwent hepatectomy from December 2000 to December 2013 were included. All pathological specimens were collected along with complete clinical and pathological data. Archived paraffin‐embedded specimens were re‐embedded into new paraffin blocks for tissue microarray (TMA). Another 57 HCC cases with portal vein embolism were recruited between August 2007 and August 2013. The follow‐up period was defined as the interval from the date of surgery to the date of death or the last follow‐up time. None of these patients had received radiotherapy or chemotherapy prior to surgery. All samples were deidentified, and all patients signed informed consents. This study was approved by the Institute Research Medical Ethics Committee of Sun Yat‐sen University Cancer Center. The study methodologies conformed to the standards set by the Declaration of Helsinki.

### 2.2 Hematoxylin and eosin and immunohistochemistry staining

TMA blocks were cut into 4‐μm slices and mounted onto glass slides. After dewaxing, slides were treated with 3% hydrogen peroxide in methanol and blocked using a biotin‐blocking kit (DAKO, Hamburg, Germany), followed by Hematoxylin and eosin and immunohistochemistry (IHC) staining. Protein expression levels of RDM1 and METTL3 were assessed by two independent pathologists (Li‐li Liu and Chun‐Hua Wang). The median IHC score was chosen as the cutoff value.

### 2.3 Cell culture

Bel‐7402, SMMC‐7721, HepG2, Bel‐7404, Huh7, and Hep3B cells, as well as the immortalized human liver cell line L‐02, were obtained from the Type Culture Collection Cell Bank, Chinese Academy of Science Committee (Shanghai, China). Cells were routinely cultured in high glucose Dulbecco’s modified Eagle’s medium (DMEM) supplemented with 10% fetal bovine serum (Gibco,Grand Island, NY, USA). Inhibitors were added to culture medium according to the manufacturer’s instructions. CHX (HY‐B1248) and PD98059 (HY‐12028) were purchased from MCE (MedChemExpress, Monmouth Junction, NJ, USA). 5‐Aza‐Cdr (A3656) was obtained from Sigma (Sigma‐Aldrich, St. Louis, MO, USA). All cells were maintained in a humidified atmosphere incubator at 37 °C and 5% CO_2_.

### 2.4 Western blot

Total protein or nuclear/cytoplasmic protein fractions were extracted from cells using lysis buffer (Beyotime Biotechnology Shanghai, China) supplemented with protease inhibitor. Western blot was performed with a standard method as previously described (Chen *et al.*, 2018b) using the following antibodies: RDM1 (1 : 500; Proteintech, Rosemont, IL, USA), p53 (1 : 2000; Santa Cruz Biotechnology, Dallas, TX, USA, β‐actin (1 : 2000; Santa Cruz), 14‐3‐3σ (1 : 1000; Santa Cruz), p21 (1 : 2000; CST, Boston, MA, USA), Cyclin A1 (1 : 1000; Santa Cruz), Cyclin B1 (1 : 1000; CST), VEGFB (1 : 1000; CST), BAX (1 : 1000; CST), RAD51 (1 : 1000; CST), hTERT (1 : 500; Abcam, UK), c‐myc (1 : 1000; Proteintech), METTL3 (1 : 1000; Abcam), METTL14 (1 : 1000; Abcam), Ras (1 : 500; CST), p‐cRaf (1 : 500; CST), ERK (1 : 1000; CST), and p‐ERK (1 : 1000; CST).

### 2.5 Quantitative real‐time RT‐PCR

Total RNA was isolated using TRIzol reagent (BIOO Scientific Co., Austin, TX, USA) according to the manufacturer’s protocol. RNA was reverse‐transcribed using M‐MLV Reverse Transcriptase (Promega Inc., Madison, WI, USA), and SYBR green‐based quantitative real‐time PCR (Vazyme Biotech, Nanjing, China) was subsequently performed. Expression analysis was performed using specific primers, and primer sequences are shown in Table S1.

### 2.6 Plasmid construction and RNA interference

Plasmids encoding RDM1 and p53 were cloned into the recombinant plasmid pcDNA 3.1/hygro (+) empty vector. METTL3 was cloned into the pENTER vector. Plasmids were transfected into HCC cell lines using Lipofectamine™ 2000 reagent (Invitrogen, Life Technologies, USA). RNAi‐mediated ablation of endogenous RDM1 was induced using small interfering RNAs (siRNAs) purchased from Santa Cruz. Other siRNAs were designed by Shanghai GenePharma Co. Ltd. (Shanghai, China) and are shown in Table S1. The specificity of siRNA sequences was confirmed by BLAST search against the human genome database. Transfection was performed using Lipofectamine™ RNAimax (Invitrogen, Life Technologies, Carlsbad, CA, USA).

### 2.7 MTT and colony formation assays

For MTT assay, 1500 cells/well were seeded into 96‐well plates with 100 μL DMEM medium containing 10% FBS. MTT solution (3 mg·mL^−1^) was added (100 μL/well) and incubated for 4 h at 37 °C for five consecutive days. After removing the supernatant, 100 μL/well DMSO was added. Absorbance was measured at 490 nm using a microplate reader. For colony formation assays, 500 cells/well were seeded into 6‐well plates and incubated for 14 consecutive days. Cells were fixed with methanol and stained with 0.1% crystal violet. Colonies were counted using imagej (NIH, Bethesda, MD USA).

### 2.8 Migration assay

Cell motility ability was assessed by Transwell assay. 3 × 10^4^ cells from each group were plated in the upper compartment of Transwell chambers (8‐μm pore size; Merck Millipore, Darmstadt, Germany) in serum‐free medium, and 1 mL fresh DMEM medium containing 15% FBS was placed in the lower chamber of a 24‐well plate. After 24‐/48‐h incubation, cells on the lower chamber were fixed using methanol and stained with 0.1% crystal violet. Cells were counted under microscopy.

### 2.9 Xenograft model

Four‐week‐old male Balb/c nude mice were purchased from Vital River Company (Beijing, China). Mice were randomized into each group and subcutaneously inoculated with HCC cells. Cells were harvested and then mixed with Matrigel (BD Biosciences, Becton Dickinson and Company, Franklin Lakes, NJ, USA) and serum‐free medium followed by subcutaneous injection. Tumor growth was monitored every three days, and mice were sacrificed four weeks after inoculation. Tumor volume was calculated using the following formula: tumor volume (mm^3^) × (length/width^2^)/2. Experimental protocols were approved by the animal institute of Sun Yat‐sen University Cancer Center according to the protocols approved by the Medical Experimental Animal Care Commission of Sun Yat‐sen University Cancer Center.

### 2.10 Immunofluorescence staining

Cells were fixed in 3.7% formaldehyde and permeabilized using 0.1% Triton X‐100 for 10 min at room temperature. After blocking in 1% bovine serum albumin, cells were incubated with diluted primary antibodies overnight at 4 °C. After washing with PBS three times, secondary antibodies were added to cells for 1 h at room temperature. DAPI solution was applied and incubated for 10 min. Images were captured using an Olympus FV1000 confocal microscope (Olympus Corporation, Tokyo, Japan).

### 2.11 Flow cytometric assay

Cells were washed with flow buffer followed by staining with propidium iodide (PI) in the dark according to the manufacturer’s instructions (Beyotime Biotechnology). Cell cycle analysis was performed using ACEA NovoCyte. Cell cycle distribution was analyzed using Beckmanculter Flow Cytoflex and modifit software (Verity, Topsham, ME, USA).

### 2.12 Immunoprecipitation (Co‐IP)

For co‐IP assays, protein was immunoprecipitated as previously described (Chen *et al.*, 2017). Briefly, total protein was harvested using co‐IP lysis buffer (Beyotime Biotechnology) supplemented with protease inhibitor (P‐8340; Sigma, St. Louis, MO, USA). The mixture was centrifuged, and the supernatant was incubated with primary antibody or negative control antibody for 4 h on ice. Twenty microliters of protein A/G plus beads (sc‐2003; Santa Cruz) was added and incubated overnight at 4 °C. Precipitates were washed three times with lysis buffer, and western blot was performed to detect the presence of the indicated protein.

### 2.13 Dual‐luciferase reporter assay

For the dual‐luciferase report assay, 293T and HepG2 cells were transfected with RDM1 promoter or mutant plasmids separately in 24‐well plates. METTL3 or empty vectors were cotransfected. A renilla luciferase vector was employed as an internal control. Luciferase activity was detected using the Dual‐luciferase Reporter Assay System Kit (Promega) and analyzed by a microplate reader.

### 2.14 m^6^A detection

Total m6A content was measured using an m6A RNA methylation quantification kit (P‐9005; Epigent) according to the manufacturer’s instructions. RDM1 m6A immunoprecipitation was performed as previously described using Magna MeRIP m6A Assay (17‐10499; Merck Millipore) (Zhang *et al.*, 2016).

### 2.15 Statistical analysis

Data are shown as the means ± standard deviation. Statistical analyses were performed with spss version 19.0 (IBM, Armonk, NY, USA) and graphpad prism (version 7.0) software (GraphPad Software Inc., La Jolla, CA, USA). Student’s *t*‐test, one‐way ANOVA, Pearson’s chi‐square test, Fisher’s exact test, Kaplan–Meier method, and multivariate Cox proportional hazards regression model were conducted according to experiments. *P* < 0.05 (two‐tailed) was considered statistically significant.

## 3 Results

### 3.1 RDM1 expression is decreased in HCC and is correlated with poor outcomes

**Figure 1:**
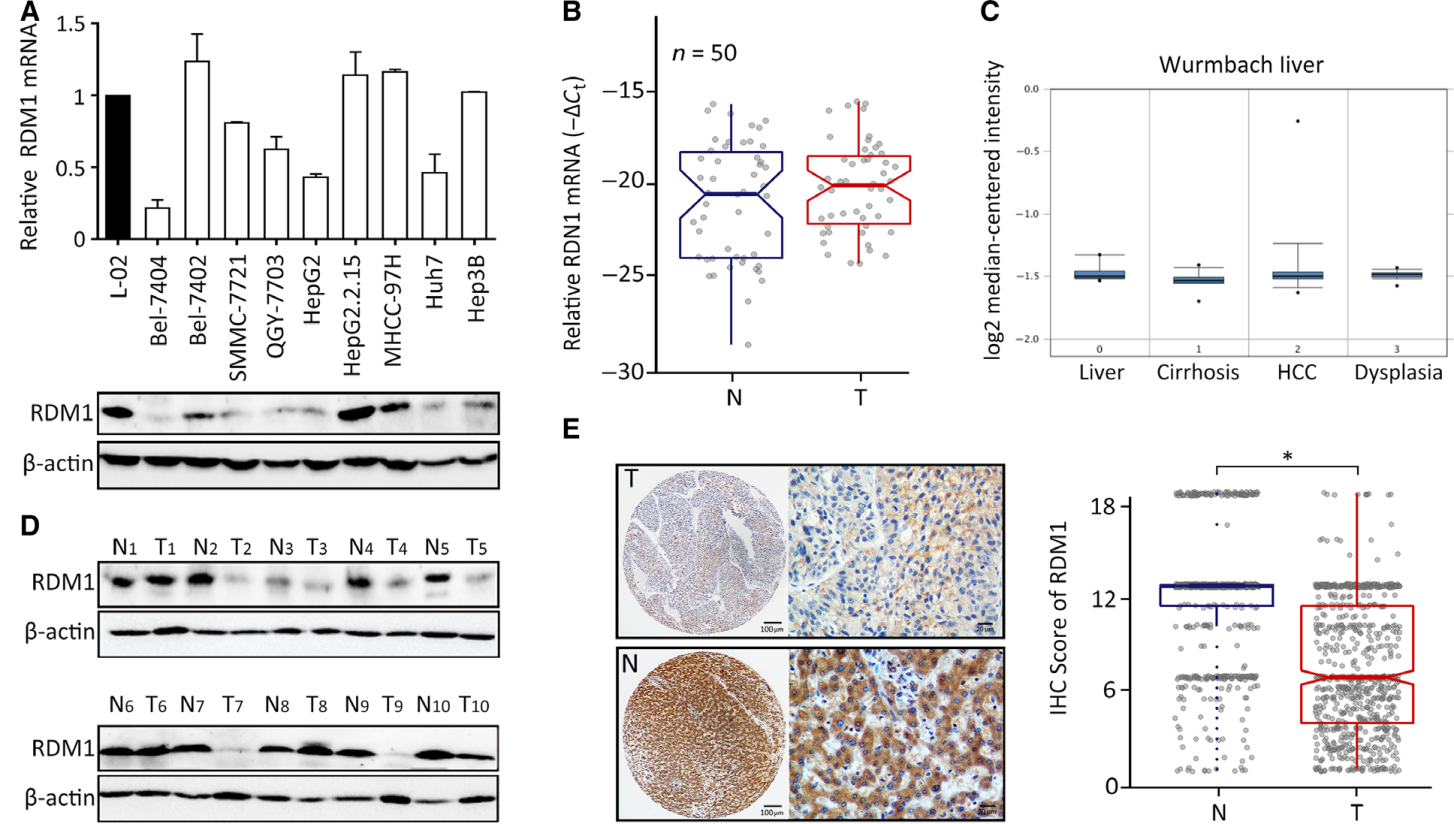
RDM1 expression is decreased in HCC. (A) The mRNA and protein expression of RDM1 in HCC cell lines and immortalized liver cell L‐02 were examined by qRT‐PCR and western blot. Statistical data were represented as mean ± SD. (B) qRT‐PCR determined RDM1 mRNA in 50 paired HCC fresh tissues and analyzed by paired *t*‐test. N, nontumor; T, tumor. (C) The expression of RDM1 mRNA was shown in Oncomine Wurmbach dataset. (D) RDM1 protein levels in 10 paired HCC and adjacent nontumor fresh tissues were shown by western blot. β‐actin was used as the indicator of the amount of loading proteins. (E) The expression of RDM1 in 755 paraffin‐embedded specimens was determined by TMA‐based IHC staining. The representative images of tumor (T) and nontumor (N) were presented, and the IHC score of each case was shown. The length of scale bars was 100 μm (left) and 20 μm (right). *P < 0.05.

To identify expression characteristics of RDM1, mRNA and protein were measured in HCC cell lines and in fresh human tissues using quantitative real‐time RT‐PCR (qRT‐PCR) and western blot assays. Compared to the immortalized hepatic cell line L‐02, RDM1 protein was reduced in most of the HCC cell lines (Fig. 1A). In 50 pairs of HCC tissues, RDM1 mRNA showed no statistical significance between tumor and nontumor samples (Fig. 1B). Consistently, Wurmbach dataset of Oncomine website revealed no RDM1 mRNA differences between normal liver, cirrhosis, HCC, and dysplasia samples (Fig. 1C). However, in 7 out of 10 HCC patients, RDM1 showed relatively low protein expression compared to matched nontumor tissues (Fig. 1D). Furthermore, IHC staining of a tissue microarray containing 755 HCC cases showed that RDM1 expression was significantly reduced in the majority of HCC specimens (446/755) (Fig. 1E).

**Figure 2:**
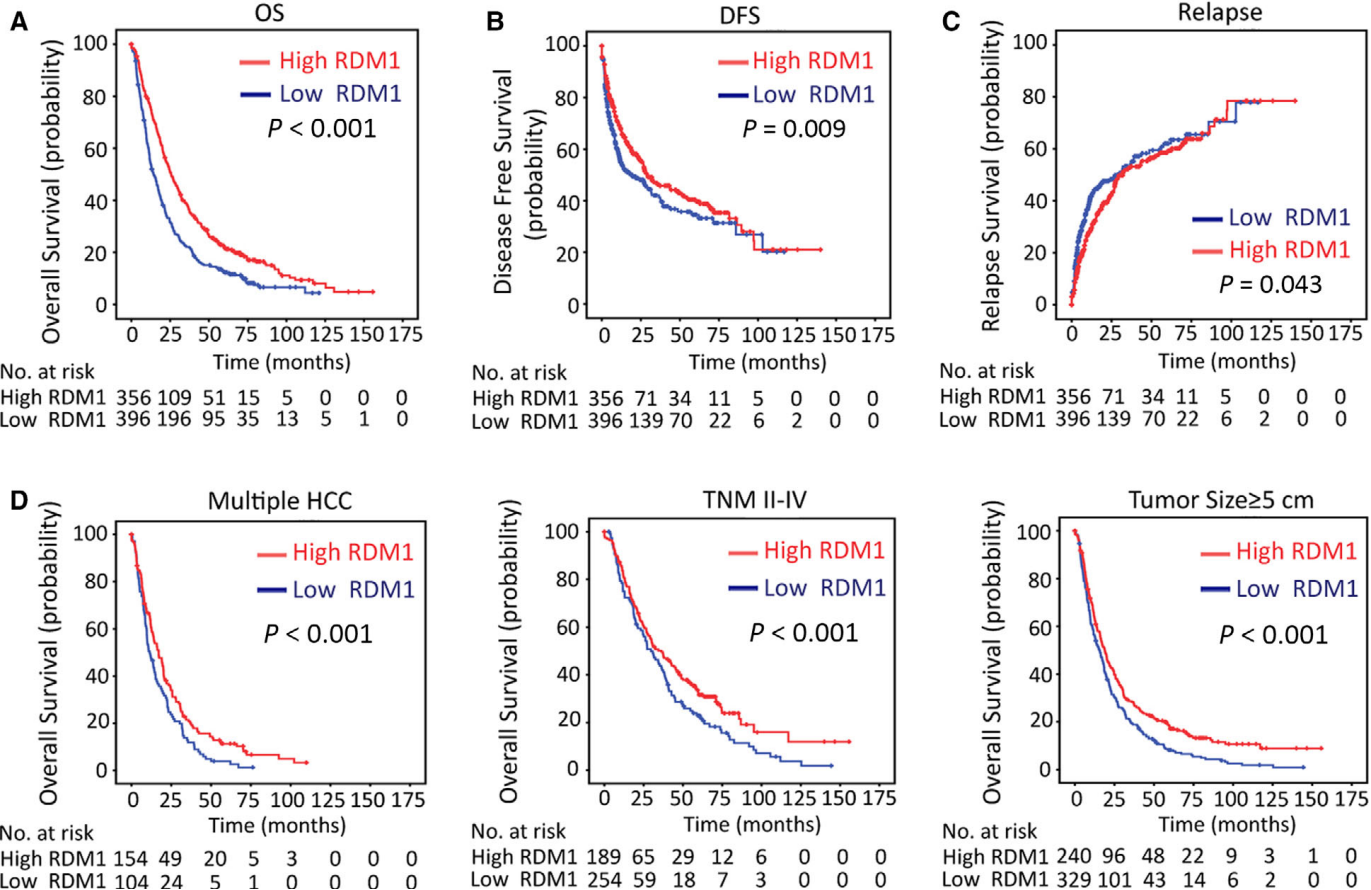
Low RDM1 expression is correlated with poor survivals. (A–C) The correlation between RDM1 expression, overall survival (A), disease‐free survival (B), and relapse (C) analysis was determined in 755 cases of TMA cohort by Kaplan–Meier survival analysis. (D) Stratified Kaplan–Meier survival analysis showed the correlation of RDM1 expression and overall survival in multiple HCC, advanced TNM stage, and large tumor size groups. Statistical data were represented as mean ± SD.

To investigate the clinical significance of RDM1 in HCC, patients were divided into relatively high and low RDM1 groups according to the median IHC score (Fig. S1). Next, we analyzed the correlation between clinical parameters and RDM1 expression. Low RDM1 expression was associated with high AFP levels, large tumor size, advanced TNM stage, and poor tumor differentiation (Table S2). Kaplan–Meier survival analysis revealed significance for low RDM1 expression in predicting unfavorable overall survival (*P* < 0.001) and disease‐free survival (*P* = 0.009) (Fig. 2A,B). Moreover, low RDM1 levels predicted recurrence of HCC (*P* = 0.043) (Fig. 2C). We further assessed the predictive value of RDM1 within clinical subgroups, and stratified analysis indicated that low RDM1 levels correlated with poor survival in multiple HCC (*P* < 0.001), advanced TNM stage (*P* < 0.001), and large tumor size subgroups (*P* < 0.001) (Fig. 2D). A multivariate Cox regression model further indicated that RDM1 was an independent prognostic factor for overall survival in HCC (Table S3). Taken together, these findings indicate that RDM1 protein, but not mRNA, was decreased in HCC and associated with unfavorable patient outcomes.

### 3.2 RDM1 suppresses cell proliferation in HCC

**Figure 3:**
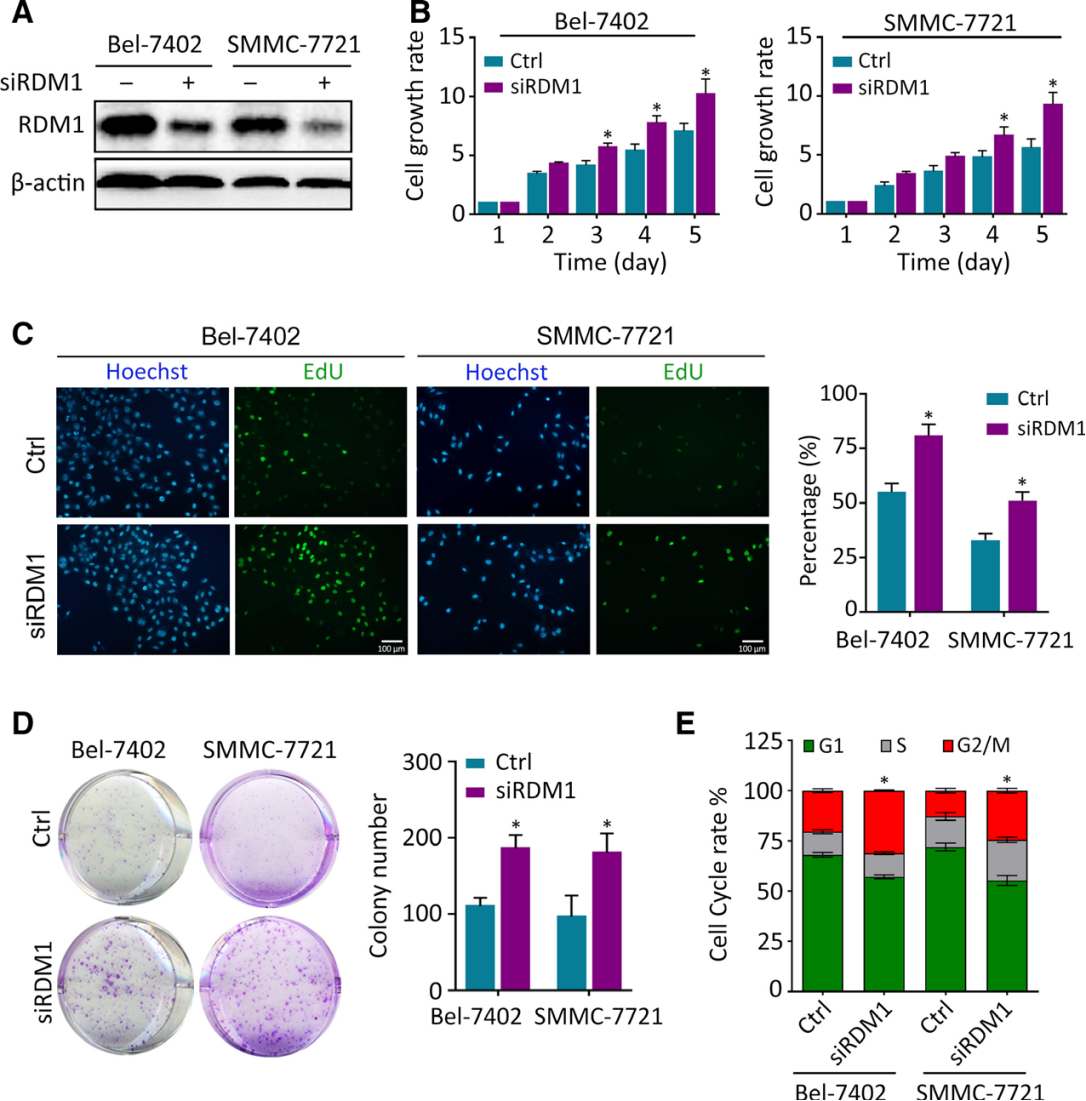
RDM1‐silencing increased cell proliferation in HCC. (A) RDM1 was silenced using siRNAs in Bel‐7402 and SMMC‐7721 cells. The transfection effect was detected by western blot. (B) Cell proliferation rates were detected by MTT assay in five consecutive days. Fold changes in each day were normalized to the absorbance measured at OD490 record in day 1. (C) EdU assays detected the DNA replication in HCC cells. The positive staining cell proportion was calculated and presented in the right panel. The length of scale bars was 100 μm. (D) Colony formation assays were used to determine the growth of cells in each group. Five hundred cells in each group were seeded into 6‐well plate, and 14 days later, the number of colonies was counted using imagej software. (E) Flow cytometric assays determined the percentage of cells in different phase of cell cycle. All the experiments were done in triplicate. Statistical data were represented as mean ± SD. Student’s *t*‐test and one‐way ANOVA methods were used to analyze the statistical difference. *P < 0.05.

To reveal the biological role of RDM1 in HCC, RDM1 was either silenced using siRNAs in Bel‐7402 and SMMC‐7721 cells or overexpressed using a pcDNA3.1‐RDM1 plasmid in HepG2 and Bel‐7404 cells (Fig. 3A and Fig. S2A). MTT assays over five consecutive days showed that cell viability was increased in siRDM1‐transfected cells and decreased in RDM1‐overexpressing cells compared with corresponding control groups (Fig. 3B and Fig. S2B). Edu assays indicate that RDM1 silencing increased, while RDM1 overexpression decreased the DNA replication proportion (Fig. 3C and Fig. S2C). The inhibiting effect of RDM1 in cell growth was further confirmed by colony formation assays (Fig. 3D and Fig. S2D). Next, we assessed the effect of RDM1 on cell cycle. Bel‐7402 and SMMC‐7721 cells were arrested at G2/M phase in response to RDM1 silencing. In contrast, RDM1 overexpression resulted in more cells at G0/G1 phase compared to the control groups (Fig. 3E and Fig. S2E).

However, alteration of RDM1 expression did not change cell migration according to Transwell assays (Fig. S3A). Moreover, RDM1 IHC staining in 57 paired HCC metastatic cases showed no expression differences between primary tumor and portal vein nodules (Fig. S3B). These results indicate that RDM1 controls cell growth and cell cycle rather than cell motility.

### 3.3 RDM1 activates p53 signaling pathway in HCC

**Figure 4:**
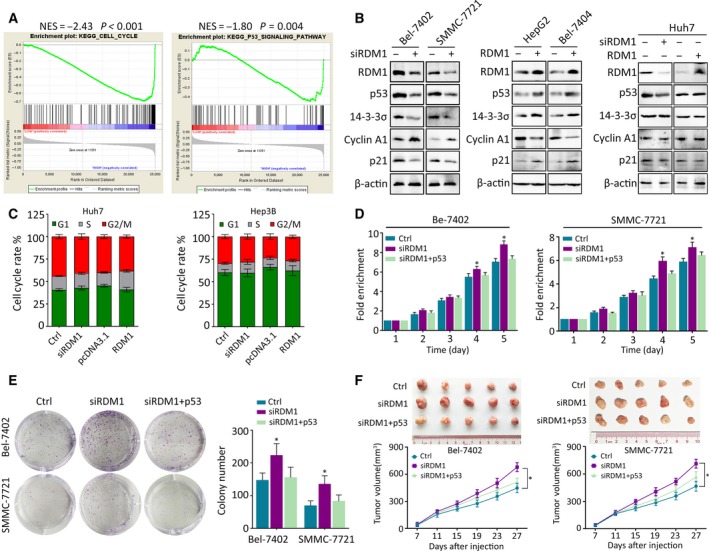
RDM1 exerts anti‐HCC activity via activation of p53 signaling pathway. (A). GSEA of HCC dataset indicated the activation of cell cycle and p53 signaling pathway of RDM1 expression obtained from TCGA. (B) The protein expression of p53 and its cell cycle‐related downstream targets were determined by western blot. (C) Huh7 and Hep3B cells were conducted with flow cytometry to detect G1, S, and G2/M phase cells in RDM1 overexpression or depleted groups. (D–F) Rescue experiments were carried out to detect the downstream effect of p53 on RDM1. p53 was overexpressed followed by silencing RDM1 in Bel‐7402 and SMMC‐7721 cells. MTT assays (D) and colony formation assays (E) showed the proliferation rate of cells in each group. *In vivo* xenograft mice experiment (F) was carried out to determine the tumor growth in nude mice. Mice were sacrificed 27 days after injecting HCC cells. The images of tumors in each group were presented, and tumor volume was calculated. All the experiments were done in triplicate. Statistical data were represented as mean ± SD. One‐way ANOVA was used to analyze the statistical difference. *P < 0.05.

Previous studies indicated that p53 is a key regulator of DNA repair and cell cycle transition. Hence, we speculated that p53 might be involved in the function of RDM1 in HCC. We conducted Gene Set Enrichment Analysis (GSEA) of a liver cancer dataset obtained from The Cancer Genome Atlas (TCGA). KEGG enrichment indicated that RDM1 is correlated with cell cycle and p53 signaling (Fig. 4A). RDM1 modulates p53 downstream targets such as p21, Cyclin A1, and 14‐3‐3σ in p53 wild‐type cell lines but not in p53‐mutated Huh7 cells (Fig. 4B). However, RDM1 had no effect on other p53 target genes such as Rad51, VEGF‐B, BAX, and c‐myc. (Fig. S4). In p53‐depleted Hep3B and Huh7 cells, neither knockdown nor ectopic overexpression of RDM1 influenced the cell cycle (Fig. 4C). These data suggest that RDM1 functions as a tumor suppressor in a p53‐dependent manner.

To validate whether RDM1 exerts anti‐HCC activities via p53, we performed rescue experiments. Restoring expression of p53 partly attenuated cell growth promoted by RDM1 depletion in Bel‐7402 and SMMC‐7721. In contrast, p53 knockdown rescued the inhibitory effect of RDM1 expression on cell proliferation in HepG2 and Bel‐7404 cells (Fig. 4D,E and Fig. S5A–C).

To further confirm the findings that RDM1 deletion is associated with progression of HCC in a p53‐dependent manner, we established a xenograft model by subcutaneously injecting HCC cells into nude mice. Tumors bearing Bel‐7402 and SMMC‐7721 cells grew faster in RDM1‐depleted groups, and this effect was inhibited by recovery of p53, while the repressive effect of RDM1 overexpression was reversed by p53 knockdown in HepG2 and Bel‐7404 cell groups (Fig. 4F and Fig. S5D).

**Figure 5:**
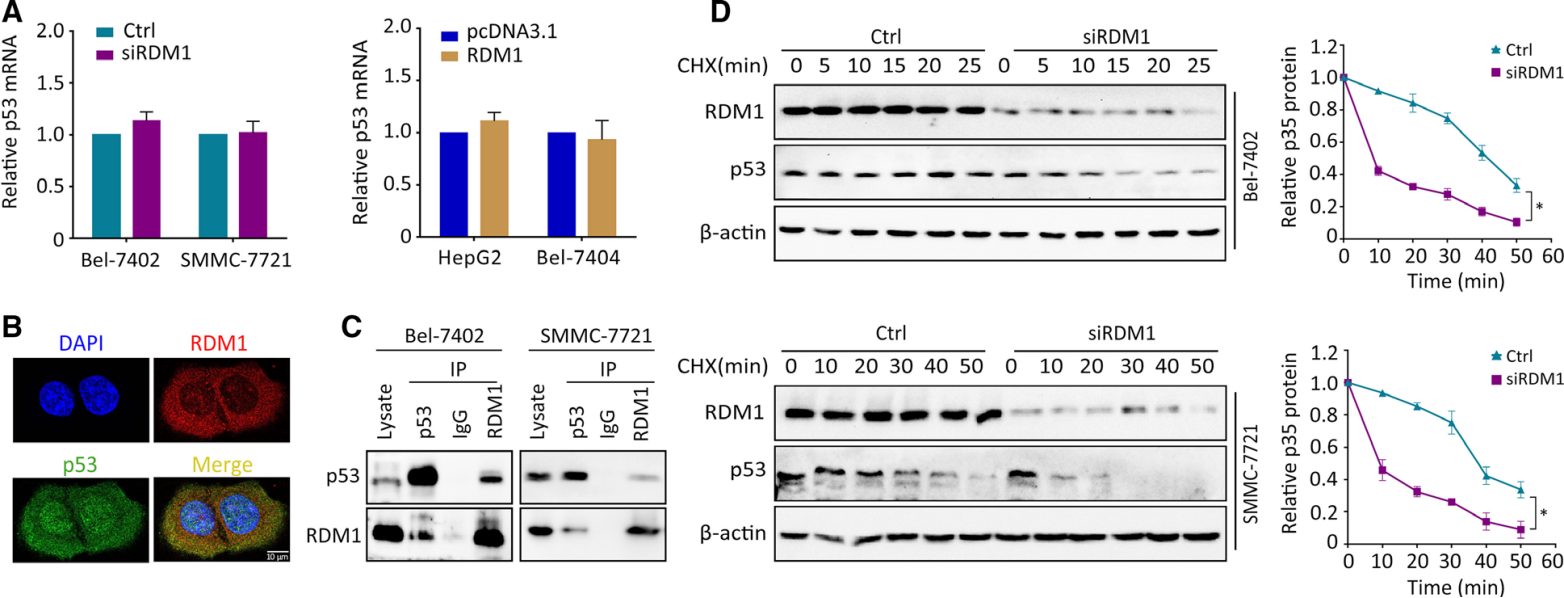
RDM1 post‐transcriptionally stabilizes p53 protein. (A) With the silencing or overexpression of RDM1, p53 mRNA levels were detected by qRT‐PCR. (B) IF staining indicating the colocalization of p53 (green) and RDM1 (red) together with DAPI (blue) in HepG2 cells. The length of scale bars was 10 μm. (C) Co‐IP assays were performed to determine the interaction of RDM1 and p53. (D) The half‐life of p53 protein was detected in RDM1‐depleted cells supplying with CHX (20 μg·mL^−1^) at different times. The total amount of p53 protein was quantitated and calculated by ImageJ software. All the experiments were done in triplicate. Student’s *t*‐test was used to analyze the statistical difference. *P < 0.05.

Next, we explored the possible mechanism by which RDM1 modulates p53. According to qRT‐PCR assays, RDM1 does not affect the transcriptional regulation of p53 in wild‐type p53 cell lines (Fig. 5A), indicating that RDM1 modulated p53 in a post‐transcriptional manner. Immunofluorescence staining showed colocalization between RDM1 and wild‐type p53 in both the cytoplasm and the nucleoplasm in HepG2 and mutated Huh7 cells (Fig. 5B and Fig. S6A). Next, co‐IP assays were performed to validate the interaction between these two proteins. The protein–protein interaction between RDM1 and wild‐type p53 in HepG2 was observed with weak interaction in Huh7 (Fig. 5C and Fig. S6B). Using the translation inhibitor CHX, we found that RDM1 overexpression markedly prolonged the half‐life of wild‐type p53 protein in HCC cells, whereas silencing RDM1 accelerated p53 degradation (Fig. 5D and Fig. S7).

### 3.4 RDM1 represses Ras/Raf/ERK signaling in the presence of p53

**Figure 6:**
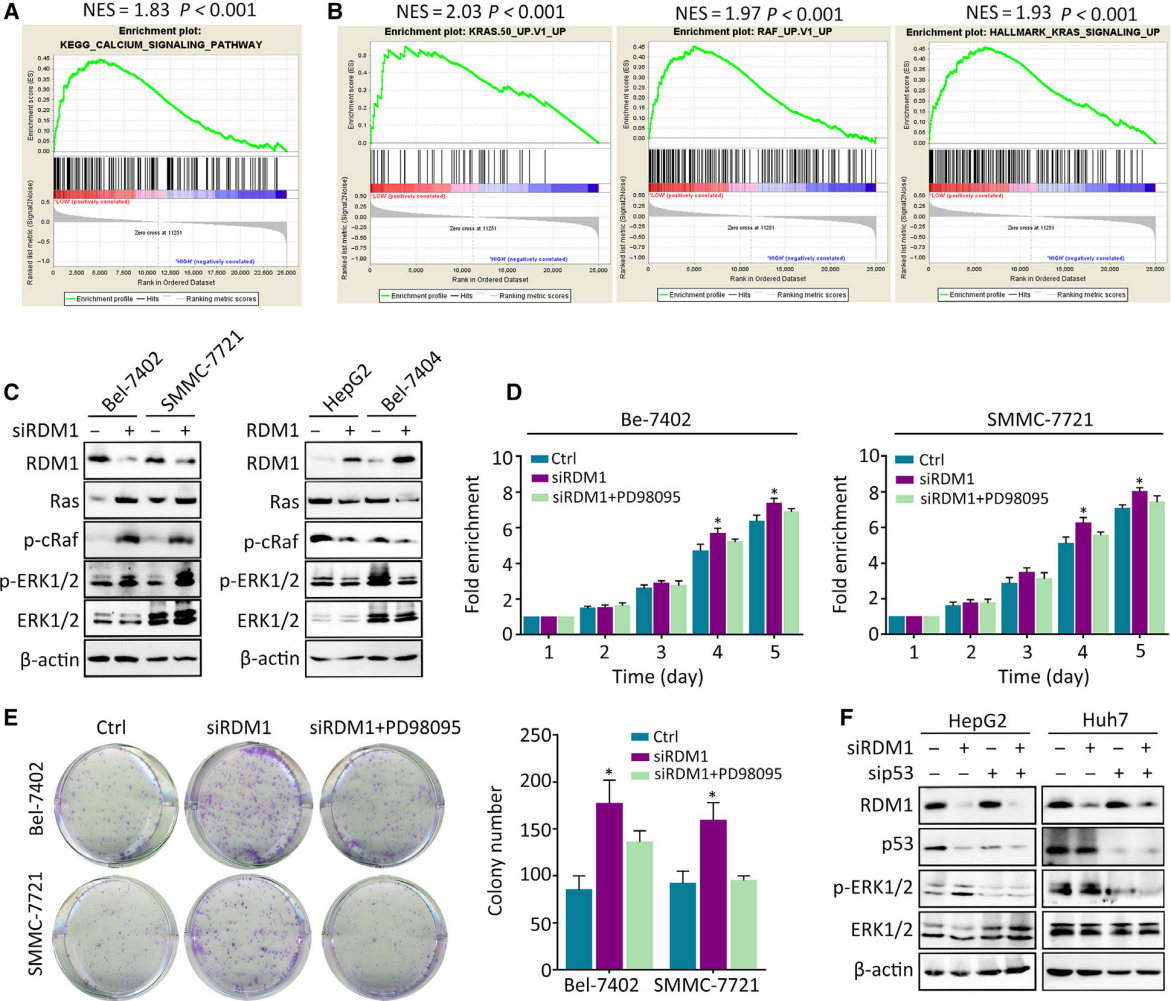
RDM1 represses Ras/Raf/ERK signaling in the presence of p53. (A, B) GSEA of HCC dataset showed the correlated of RDM1 expression obtained from TCGA with related pathways. (C) Western blot validated the differential expression of Ras/Raf/ERK signaling pathway in RDM1 overexpression or depleted cells. (D, E) MTT assay (D) and colony formation assays (E) indicated the cell growth in RDM1‐depleted or ERK inhibitor PD98095‐treated groups. (F) Western blot detected the expression of p‐ERK with the depletion of p53 and RDM1 in HepG2 and Huh7 cells. All the experiments were done in triplicate. One‐way ANOVA method was used to analyze the statistical difference. *P < 0.05.

According to GSEA, DNA repair‐related datasets (DNA replication, base excision, mismatch repair, and nucleotide excision repair) were altered in high RDM1 groups, as it is a key regulator in DNA double‐strand break repair and recombination (Fig. S8). To unveil the underlying molecular mechanism controlled by RDM1 in addition to its canonical function, we conducted transcriptome sequencing. The most altered pathway according to *P*‐values both in Bel‐7402 and SMMC‐7721 cells treated with siRDM1 was mineral absorption (Table S4). In this pathway, metal ions, such as Ca^2+^, Cu^2+^, and Zn^2+^, are essential second messengers involved in cancer progression. GSEA validated enriched calcium signaling and oncological pathways KRAS and RAF in low RDM1 groups (Fig. 6A,B). Given that Ras/Raf is upstream of the MAPK pathway that results in ERK phosphorylation, we hypothesized that RDM1 modulated HCC progression by suppressing KRAS‐mediated MAPK/ERK signaling. To test this hypothesis, we measured the expression of p‐c‐raf and p‐ERK1/2 in HCC cells bearing different RDM1 expressing status. Knockdown of RDM1 resulted in elevated p‐c‐raf and p‐ERK1/2 levels Bel‐7402 and SMMC‐7721 cells, while in HepG2 and Bel‐7404 cells, RDM1 overexpression suppressed levels of p‐c‐raf and p‐ERK1/2 (Fig. 6C). Next, we conducted rescue experiments by treating cells with the ERK inhibitor PD98095. As expected, cell growth induced by RDM1‐silencing was attenuated in response to PD98095 treatment (Fig. 6D,E). Furthermore, regulation of p‐ERK1/2 by RDM1 requires the presence of wild‐type p53 instead of mutated p53 (Fig. 6F). These findings suggest that the Ras/Raf/ERK signaling pathway plays a critical role in RDM1‐mediated HCC progression.

### 3.5 RDM1 is inhibited by METTL3‐mediated m^6^A modification

**Figure 7:**
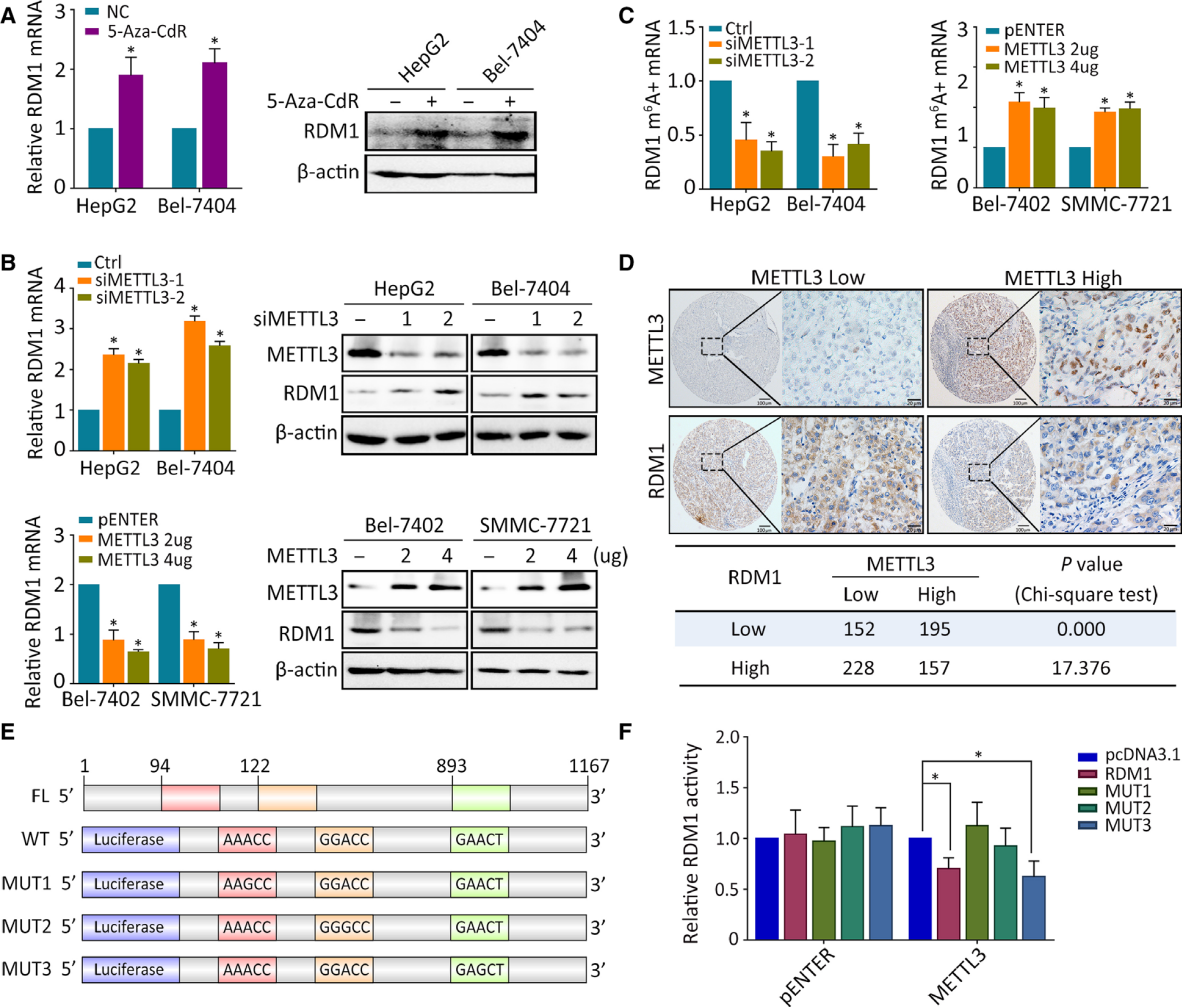
RDM1 is inhibited by METTL3‐mediated m^6^A modification. (A) RDM1 mRNA and protein expression levels were tested in HepG2 and Bel‐7404 cells treated with 5 μm 5‐Aza‐Cdr for 24 h. (B) qRT‐PCR and western blot assays showed the mRNA and protein expression of RDM1 in METTL3 knockdown cells (upper) and METTL3 overexpression cells (down). (C) m^6^A immunoprecipitation and qRT‐PCR were performed to determine the relative percentage of RDM1 mRNA with methylation. (D) IHC staining of RDM1 and METTL3 in TMA was presented, and their correlation was calculated. The length of scale bars was 100 μm (left) and 20 μm (right) (E) The schematic graph of RDM1 possible m^6^A modification region and mutants were cloned to pGL3‐Basic reporter. (F) Luciferase assays were carried out to identify the m^6^A modification region in RDM1 catalyzed by METTL3. All the experiments were done in triplicate. Student’s t‐test and one‐way ANOVA methods were used to analyze the statistical difference. *P < 0.05.

Epigenetic silencing is the most frequent method of gene silencing. Upon demethylase 5‐Aza‐CdR treatment, RDM1 protein levels increased (Fig. 7A). By analyzing 2000 bp upstream of the RDM1 promoter using the website http://www.urogene.org/methprimer/, we did not identify specific CpG islands, indicating that DNA methylation might not be the cause of RDM1 downregulation in HCC (data not shown). As mRNA methylation leads to gene suppression, we analyzed the effect of two RNA methyltransferases, METTL3 and METTL14, on RDM1. Silencing of METTL3 increased, while overexpression of METTL3 decreased, RDM1 expression (Fig. 7B). However, METTL14 had no effect on RDM1 expression (Fig. S9A). Quantitative analysis of total m^6^A in HepG2 and Bel‐7404 cells indicated that METTL3 depletion decreased, while overexpression increased, total m^6^A mRNA and m^6^A modified RDM1 mRNA (Fig. S9B and Fig. 7C). An inverse correlation between METTL3 and RDM1 mRNA was identified by qRT‐PCR in 50 paired tissues (Fig. S9C). Furthermore, TMA‐based IHC validated that 59.2% of patients exhibited decreased RDM1 in the high METTL3 expression group (Fig. 7D and Fig. S9D). To identify possible target sites of METTL3 on RDM1, we analyzed the RDM1 sequence from 3′UTR to 5′UTR to uncover possible methylation sites in the CDS region (Fig. 7E). We constructed wild‐type and mutant CDS plasmids of RDM1 for dual‐luciferase assay to test specific modifying sites. MUT1 and MUT2, but not MUT3, sites were modulated by METTL3 (Fig. 7F). Taken together, these results indicate that RDM1 is methylated by METTL3, leading to its decrease in HCC.

## 4 Discussion

Hepatocellular carcinoma is one of the most lethal tumors, with a complicated regulatory network. There is a great need to improve our understanding of the molecular mechanisms underlining HCC development due to the currently limited available therapies. In this study, we demonstrated that RDM1 functions as a tumor suppressor in HCC by inhibiting cell proliferation but not migration. Clinically, we identified an association between low RDM1 and poor patient outcome, suggesting RDM1 as a promising prognostic and therapeutic factor in HCC.

Previous studies have revealed that RDM1 promotes cancer growth by inducing G2/M cell cycle arrest (Li *et al.*, 2017a; Tong *et al.*, 2018). In line with these studies, we demonstrated that RDM1 influenced cell cycle in HCC in a p53‐dependent manner. The interaction between RDM1 and wild‐type p53, which leads to p53 stability, was further identified in HCC cell lines. Since p53 is the most frequently mutated gene in HCC according to TCGA, the correlation between RDM1 and gain‐of‐function or loss‐of‐function p53 mutations should not be ignored. In p53‐mutated Huh7 and p53‐null Hep3B cell lines, RDM1 failed to regulate the cell cycle. These results indicate that RDM1 regulates HCC cells bearing different p53 status using a diverse range of signaling molecules.

In contrast to our findings, negative regulation of p53 by RDM1 was observed in lung cancer (Tong *et al.*, 2018). According to previous studies, increased turnover of p53 is responsible for TGF‐β1 effects in both human HaCaT cells and mouse LTR‐6 myeloblastic cells (Landesman *et al.*, 1997). However, TGF‐β promotes p53 stability in U2OS and HEK293 cells (Liu *et al.*, 2017). PKM2 differentially regulates p53 depending on its redox status: Reduced tetrameric PKM2 enhances p53 transcriptional activity, while oxidized tetrameric (but not monomeric) PKM2 suppresses p53 transcriptional activity (Saleme *et al.*, 2019). Moreover, HIF‐1α upregulates p53 expression in HK‐2 cells (Liu *et al.*, 2018), while suppressing p53 expression in the context of irradiation in HeLa cells (Fu *et al.*, 2015). These results indicate that p53 is differentially regulated by diverse genes in various cell types. Hence, we speculate that the divergent regulation pattern between our study and Tong *et al.* (2018) might be due to the different status of RDM1 since it has been reported to have multiple splice variants shuttled from the nucleus to the cytoplasm. Another possible reason for these differences could be attributed to the differentially expressed ubiquitin‐related enzymes related to p53 turnover (Brooks and Gu, 2011). For example, COP1 was reportedly overexpressed in HCC and decreased in lung cancer according to Oncomine datasets (Lee *et al.*, 2010). Whether RDM1 cofunctions with COP1 to differentially regulate p53 requires further study.

TP53 mutations result in loss of wild‐type functions or acquire new oncogenic properties (Muller and Vousden, 2014). For example, Zheng et al reported that knocking down SIRT1 led to the upregulation of PTEN‐PI3K‐AKT pathway in p53 wild‐type cell line HepG2 and this effect was not observed in p53‐mutated cell line PLC5 cells (Zhang *et al.*, 2015). Lim SO et al indicated that Notch1 and Snail/NICD expression was correlated with p53 expression in wild‐type p53 cells but not elevated in p53‐mutated or knockout cells (Lim *et al.*, 2011). These results indicated that the p53 exert different roles in tumor cells depending on its function. According to documentations, Huh7 harbors Y220C mutation within DNA‐binding region of p53. This point mutation endowed p53 with oncogenic ability, leading to p53 cytoplasm accumulation and destabilization (Baud *et al.*, 2018; Iwao and Shidoji, 2014). p53^Y220C^ was p21 defective but retains the function of Cyclin B (Wu *et al.*, 2013), which is concordant with our results. We assume that the damaged transcriptional function of p53^Y220C^ partially accounts for the differential expression of p53 downstream targets modulated by RDM1.

Our findings also revealed dysregulation of cancer‐related minerals, including Ca^2+^, Zn^2+^, and Cu^2+^ et al. GSEA indicated enrichment of Ca^2+^ in the low RDM1 group. Ca^2+^ is a ubiquitous second messenger for many cellular processes, including apoptosis (Orrenius *et al.*, 2003), epithelial‐to‐mesenchymal transition, and therapeutic resistance (Monteith *et al.*, 2017). The intracellular calcium pathway is inactivated or Ca^2+^ intake is impaired in cancer progression (Monteith *et al.*, 2007; Yang *et al.*, 2018). p53 had been implicated in the regulation of Ca^2+^‐dependent pathways (Can *et al.*, 2013; Giorgi *et al.*, 2015). Meanwhile, the Ras/Raf/ERK pathway was proven to be associated with Ca^2+^ aberration (Kupzig *et al.*, 2005; Zhang *et al.*, 2009). These data indicate that p53 and Ras/Raf/ERK are both involved in downstream effects induced by RDM1. We demonstrated that loss of RDM1 promotes tumor growth through activation of p53 and Ras/Raf/ERK pathways. However, how Ca^2+^ is involved in the function of RDM1 and the progression of HCC requires further investigation.

Recent studies have focused on reversible methylation of m^6^A mRNA modification, which leads to downregulation of multiple tumor suppressor genes. Distinct from cofactors METTL14 and WTAP, which were found to be restricted to the nuclear fraction, METTL3 was detectable in the cytoplasm, indicating its function in translational regulation was independent of its catalytic activity (Wang *et al.*, 2014). The role of METTL3 in cancer is controversial. METTL3 acts as a tumor suppressor in renal cell carcinoma (Li *et al.*, 2017b), while METTL3 promotes chemo‐ and radioresistance in pancreatic cancer cells and promotes breast cancer progression (Cai *et al.*, 2018; Taketo *et al.*, 2018). In liver cancer, METTL3 is frequently upregulated in human HCC and contributes to HCC progression (Chen *et al.*, 2018a). In line with these results, METTL3 acted as an onco‐protein that suppressed the tumor suppressor RDM1 because m^6^A modification results in splicing of mRNA, which regulates gene expression (Dominissini *et al.*, 2012; Louloupi *et al.*, 2018). Whether METTL3 sequesters the stability of RDM1 mRNA or induces variable mRNA splicing remains for future investigation.

## 5 Conclusions

Our study identified a novel tumor suppressor gene RDM1 in HCC. Loss of RDM1 correlates with unfavorable clinical outcomes, promotes HCC cell proliferation, and induces G2/M cell cycle arrest. The newly identified METTL3/RDM1/p53/ERK axis provides potential prognostic and therapeutic targets for HCC treatment.

## Conflict of interest

The authors declare no conflict of interest.

## Author contributions

S‐LC, CZZ, and J‐PY conceived and designed the study; S‐LC, L‐LL, C‐HW, and XY generated, collected, assembled, and analyzed the data; C‐HW, S‐XL and Y‐FH scored and evaluated the IHC‐stained slides; S‐LC, CZZ, and J‐PY drafted and revised the manuscript; and all authors made approval of the final version of the manuscript.

## Supporting information


**Fig. S1.** IHC staining of RDM1 in HCC TMA.
**Fig. S2.** RDM1 suppresses cell proliferation in HCC.
**Fig. S3.** RDM1 has no impact on cell migration.
**Fig. S4.** The expression of p53 down‐stream targets modulated by RDM1.
**Fig. S5.** p53 is the downstream target of RDM1.
**Fig. S6.** The localization and interact between RDM1 and mutated p53.
**Fig. S7.** RDM1 elongated the half‐life of p53 protein.
**Fig. S8.** GSEA analysis of RDM1.
**Fig. S9.** METTL3 suppressed the expression of RDM1.

**Table S1.** siRNAs and Primers.
**Table S2.** Correlation of clinicopathological parameters and RDM1 expression.
**Table S3.** Univariate and multivariate analyses of clinicopathological and RDM1 expression for overall survival in overall cohort.

**Table S4.** KEGG Enrichment in Bel‐7402 and SMMC‐7701 cell lines NC Vs. siRDM1.
