# Supplementary material for: Loss of RDM1 enhances hepatocellular carcinoma progression via p53 and Ras/Raf/ERK pathways
Source: Mol Oncol. 2019 Dec 19;14(2):373–86. doi: 10.1002/1878-0261.12593 (PMC6998392; doi:10.1002/1878-0261.12593)
Supplement: Supplementary file 1 — Fig. S1. IHC staining of RDM1 in HCC TMA. Fig. S2. RDM1 suppresses cell proliferation in HCC. Fig. S3. RDM1 has no impact on cell migration. Fig. S4. The expression of p53 down‐stream targets modulated by RDM1. Fig. S5. p53 is the downstream target of RDM1. Fig. S6. The localization and interact between RDM1 and mutated p53. Fig. S7. RDM1 elongated the half‐life of p53 protein. Fig. S8. GSEA analysis of RDM1. Fig. S9. METTL3 suppressed the expression of RDM1. [file MOL2-14-373-s001.docx]

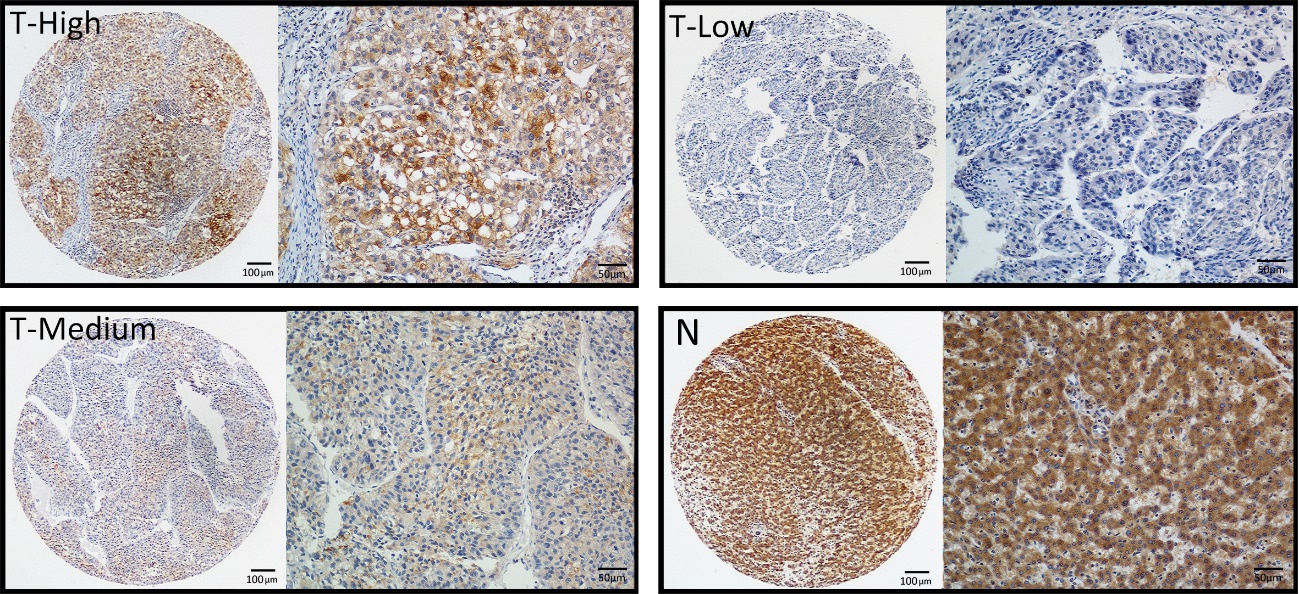


**Supplementary Figure 1: IHC staining of RDM1 in HCC TMA**

Representative images of RDM1 IHC staining presented the high, medium and low intensity for tumor or nontumor tissues in HCC TMA. The length of scale bars was 100 μm (left) and 50 μm (right). T=Tumor, N=nontumor.


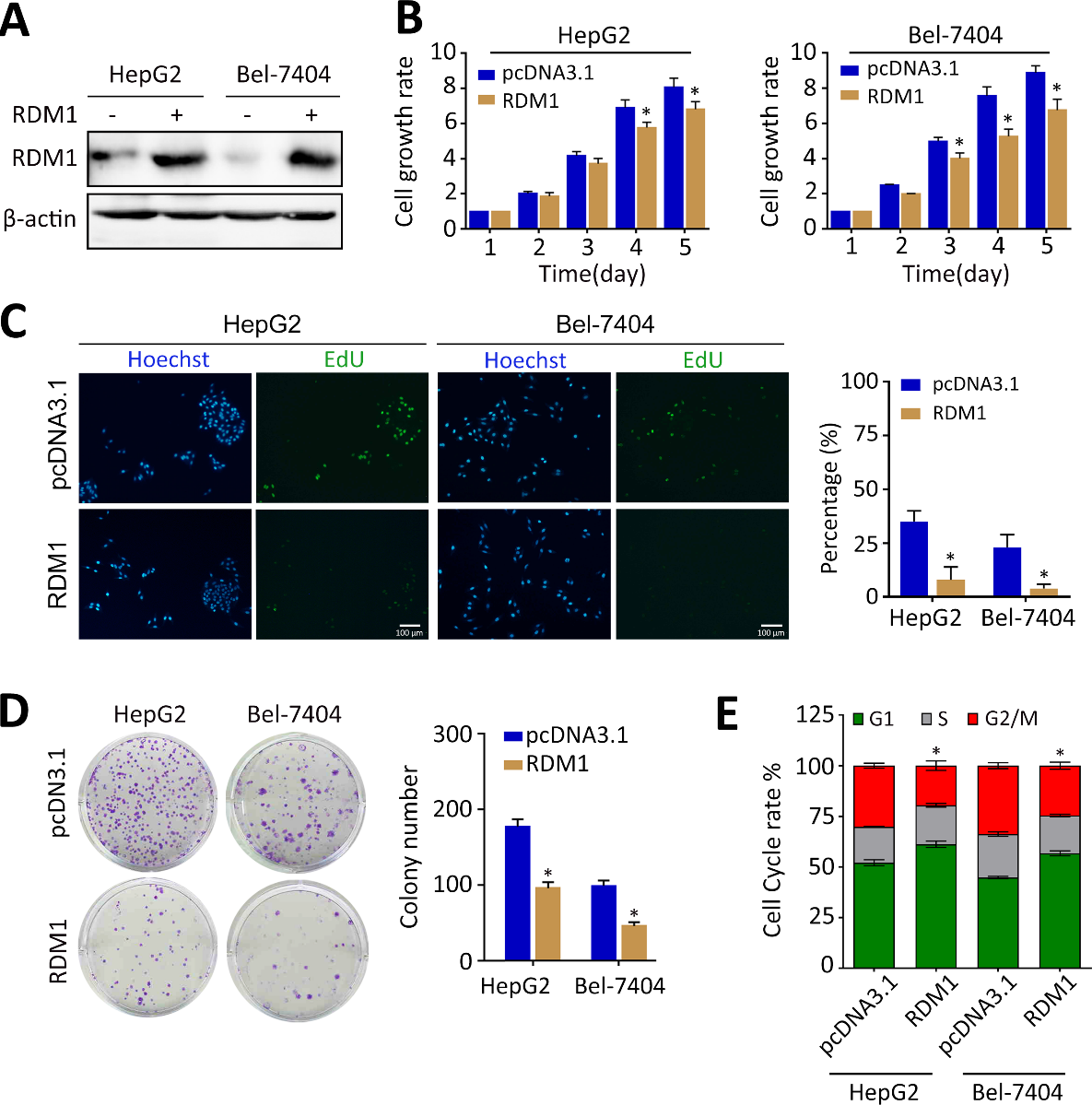


**Supplementary Figure 2. RDM1 suppresses cell proliferation in HCC**

**A.** RDM1 was overexpressed in HepG2 and Bel-7404 cells. The transfection effect was detected by Western blot. **B.** Cell proliferation rates were detected by MTT assay in five consecutive days. Fold changes in each day were normalized to the absorbance measured at OD490 record in day 1. **C.** Edu assays detected the DNA replication in HCC cells. The positive staining cell proportion were calculated and presented in the right panel. The length of scale bars was 100 μm. **D.** Colony formation assays were used to determine the growth of cells in each group. 500 cells in each group were seeded into 6-well plate and 14 days later, the number of colonies were counted using Image J software. **E.** Flow cytometry assays determined the percentage of cells in different phase of cell cycle. Statistical data were represented as mean± SD. Student’s t-test and one-way ANOVA methods were used to analyzed the statistical difference. **P*<0.05.


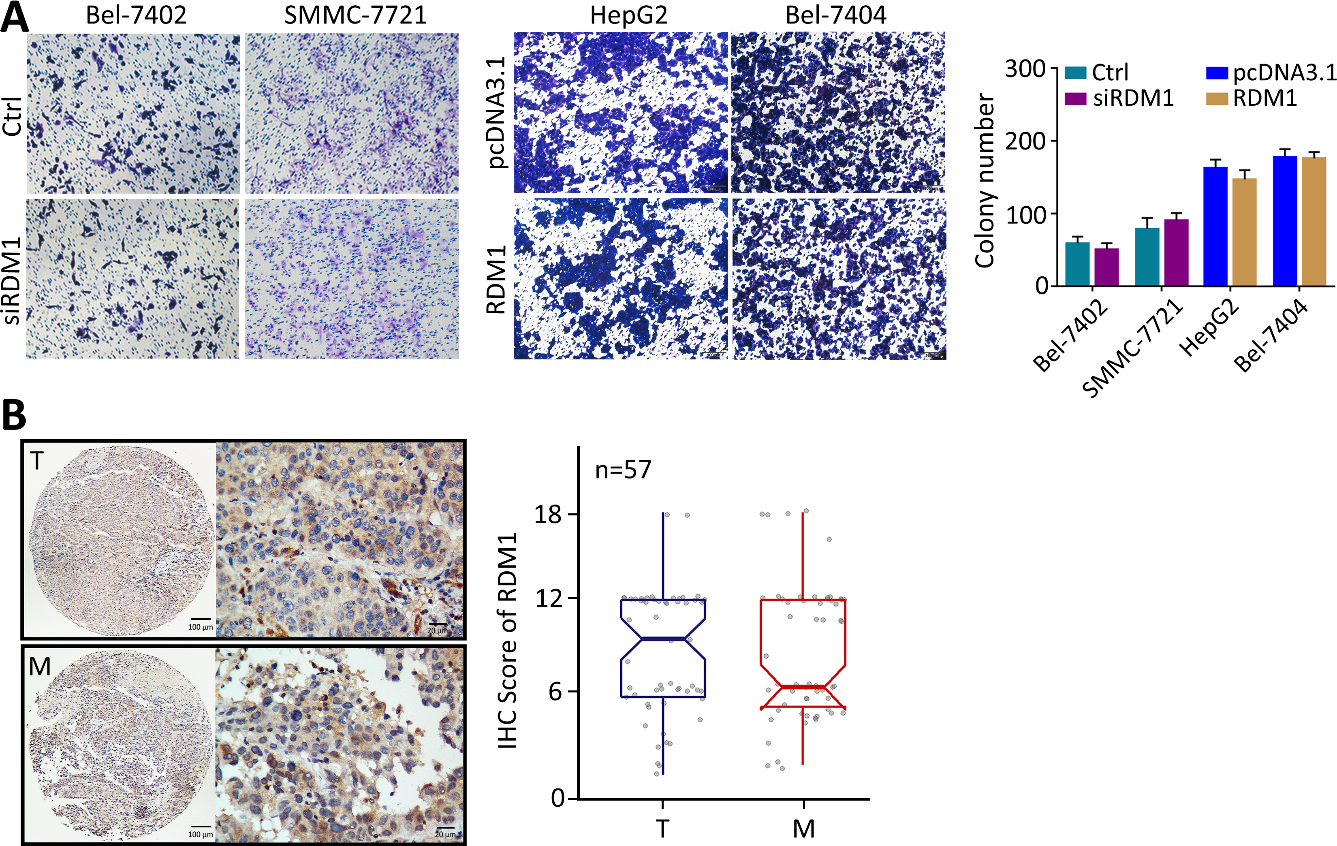


**Supplementary Figure 3: RDM1 has no impact on cell migration**

**A.** Transwell migration assays indicated the movement ability of cells in RDM1-overexpressed and RDM1-silenced groups. No statistical difference was detected between indicated groups. **B.** IHC staining of RDM1 was conducted in TMA cohort of 57 HCC patients with primary tumor (T) and portal vein metastatic nodules (M). Statistic data of IHC scores in each case were shown. The length of scale bars was 100 μm (left) and 20 μm (right).


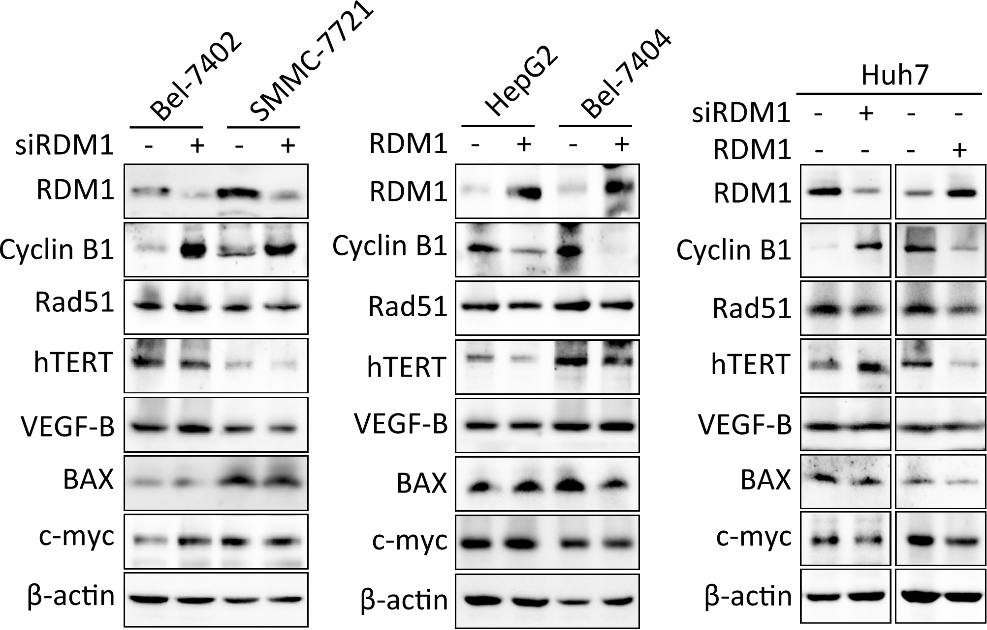


**Supplementary Figure 4: The expression of p53 down-stream targets modulated by RDM1**

The expression of p53 down-stream targets were detected by Western blot in RDM1 silencing or overexpression in HCC cell lines.


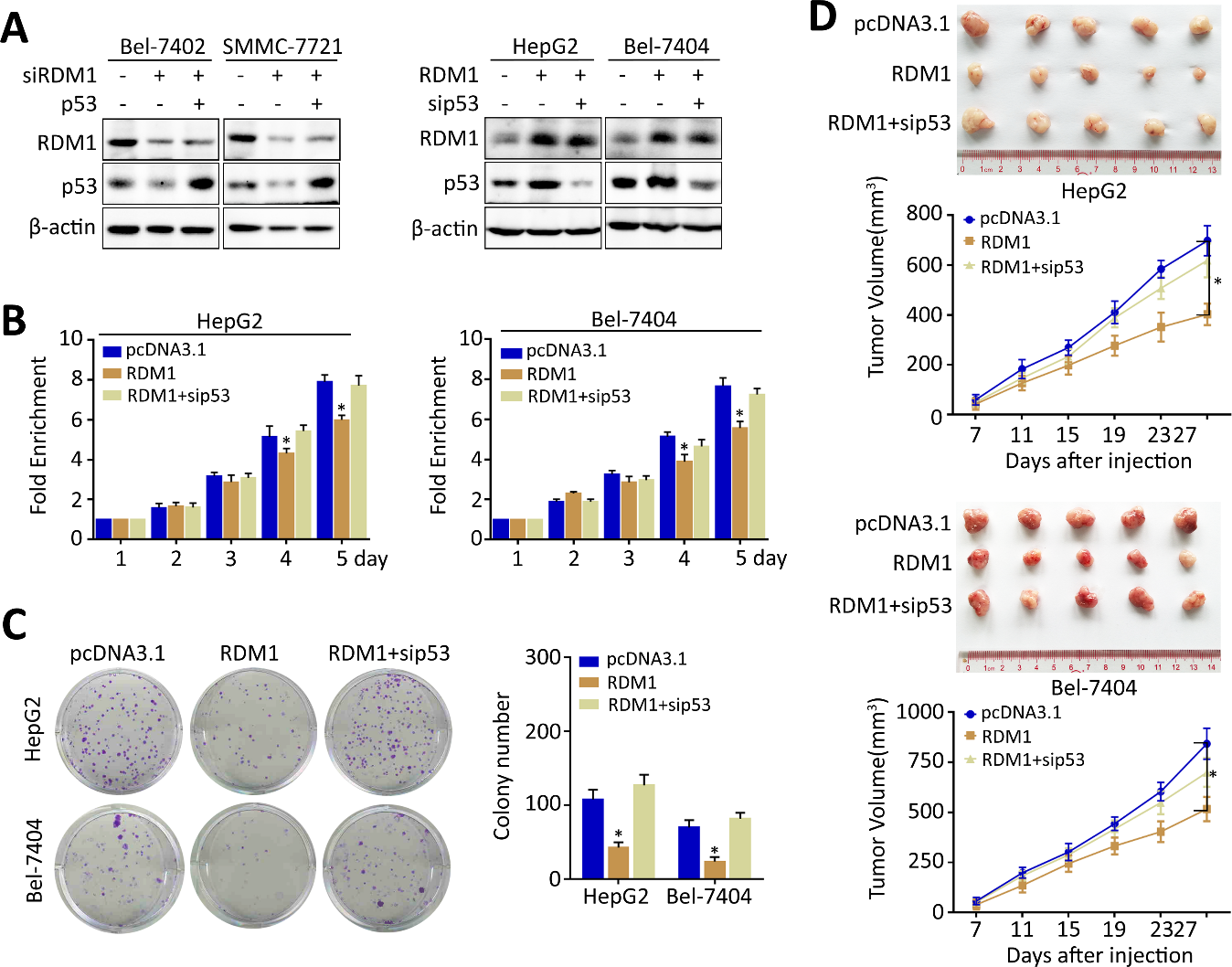


**Supplementary Figure 5: p53 is the downstream target of RDM1**

**A.** The expression of RDM1 and p53 were detected by Western blot in rescue experiments. **B-D.** Rescue experiments were carried out to detect the downstream effect of p53 on RDM1. p53 was silenced followed by RDM1 overexpression in HepG2 and Bel-7404 cells. MTT (B), colony formation (C) and *in vivo* assays (D) showed the proliferation rate of cells in each group. Statistical data were represented as mean± SD. One-way ANOVA was used to analyzed the statistical difference. * *P*<0.05.


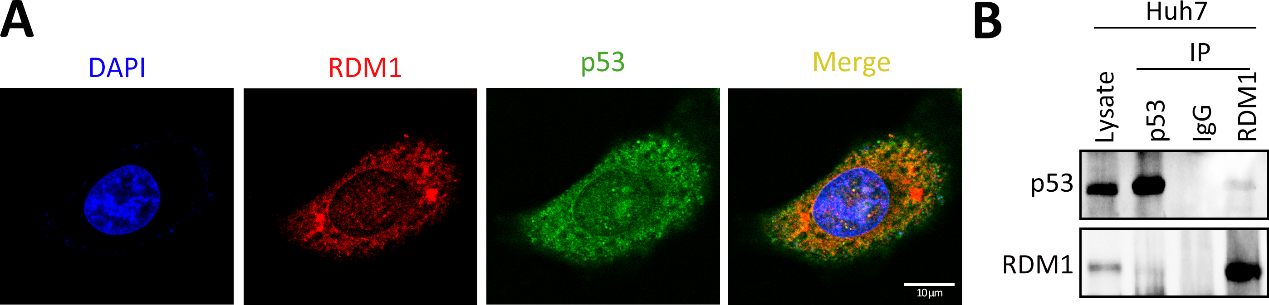


**Supplementary Figure 6: The localization and interact between RDM1 and mutated p53**

**A.** IF staining showed the subcellular localization of RDM1 and p53 in Huh7 cell. The length of scale bars was 10 μm. **B.** IP assay indicated the interaction between RDM1 and mutated p53.


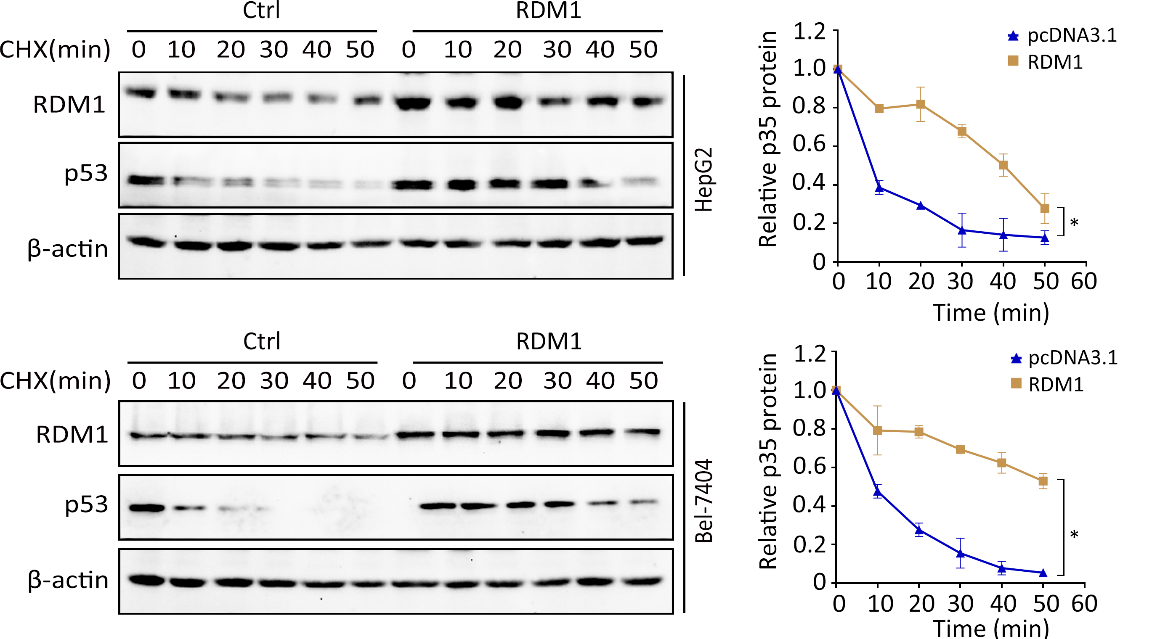


**Supplementary Figure 7: RDM1 elongated the half-life of p53 protein**

The half-life of p53 protein were detected in HepG2 and Bel-7404 with RDM1 overexpression and CHX (20 μg/ml) supplement for different times. The degradation rates of p53 protein were calculated by Image J software. Statistical data were represented as mean± SD. Student’s t-test was used to analyzed the statistical difference. **P*<0.05.


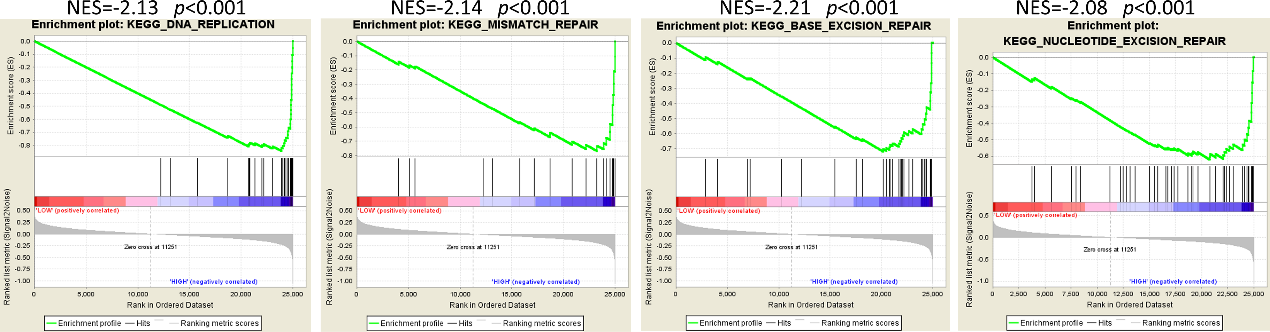


**Supplementary Figure 8: GSEA analysis of RDM1**

RDM1 mRNA expression of HCC was obtained from TCGA. Cases were divided into high and low RDM1 expression groups according to median value. GSEA analysis was conducted according to protocols in http://software.broadinstitute.org/gsea/index.jsp.

**
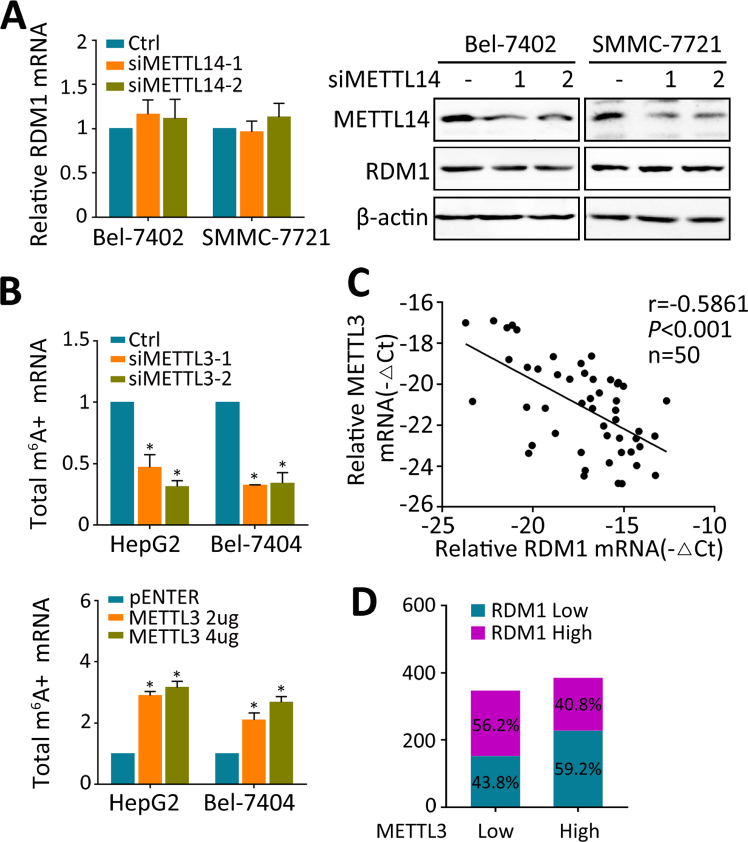
**

**Supplementary Figure 9: METTL3 suppressed the expression of RDM1**

**A.** RDM1 mRNA was detected by qRT-PCR and protein was detected by western blot with the silencing of METTL14. **B.** METTL3 was silenced or overexpressed and total mRNA m6A modification level was detected by Epigentak P-9005 kit. **C.** The correlation between RDM1 and METTL3 mRNA was analyzed in 50 paired HCC tissues. **D.** The percentage of RDM1 cases in METTL3 low and high expression groups according to IHC staining were shown. Statistical data were represented as mean± SD. Student’s t-test and one-way ANOVA methods were used to analyzed the statistical difference. **P*<0.05.
