# Supplementary material for: Loss of RDM1 enhances hepatocellular carcinoma progression via p53 and Ras/Raf/ERK pathways
Source: Mol Oncol. 2019 Dec 19;14(2):373–86. doi: 10.1002/1878-0261.12593 (PMC6998392; doi:10.1002/1878-0261.12593)
Supplement: Supplementary file 2 — Table S1. siRNAs and Primers. Table S2. Correlation of clinicopathological parameters and RDM1 expression. Table S3. Univariate and multivariate analyses of clinicopathological and RDM1 expression for overall survival in overall cohort. [file MOL2-14-373-s002.doc]

**Table S1. siRNAs and Primers**

siRNAs

|  | **Identifier Type** | **Sense sequence(5’-3’)** |  |  | |
| --- | --- | --- | --- | --- | --- |
|  | p53-1 | CCACUUGAUGGAGAGUAUU |  |  | |
|  | p53-1  RDM1-1  RDM1-2  RDM1-3  METTL3-1  METTL3-2 | TGCGTGTGGAGTATTTGGATG CUAUGCCGUCAUUAAGUUUtt  GAACUGGCGAAUUACUACUtt  GUGUUGCCAUCCUGUGAUUtt  GCAAGUAUGUUCACUAUGATT  CTGCAAGTATGTTCACTATGA |  |  | |
|  | METTL14-1  METTL14-2 | AAGGATGAGTTAATAGCTAAA  TGGTGCCGTGTTAAATAGCAA |  |  | |
| Primers   | **Identifier Type** | **Sequence (5’-3’)** |  | | --- | --- | --- | | RDM1-F | GCCCATCCTGGTTTCTATGCC |  | | RDM1-R | AGACGAACCTTGACTGGAGAT |  | | METTL3-F | CAAGCTGCACTTCAGACGAA |  | | METTL3-R | GCTTGGCGTGTGGTCTTT |  | | 18S-F | TGAGAAACGGCTACCACATCC |  | | 18S-R | ACCAGACTTGCCCTCCAATG |  | | | | | |  |

**Table S2. Correlation of clinicopathological parameters and RDM1** expression

| Variable | RDM1 expression | | | |
| --- | --- | --- | --- | --- |
|  | All cases | Low expression | High expression | *P* value^a^ |
| Age (years) ^b^ |  |  |  | 0.060 |
| < 49 | 364 | 186(51.1%) | 178 (48.9%) |  |
| ≥ 49 | 391 | 173 (44.2%) | 218 (55.8%) |  |
| Gender |  |  |  | 0.620 |
| Male | 665 | 314(47.2%) | 351 (52.8%) |  |
| Female | 90 | 45 (50.0 %) | 45 (50.0%) |  |
| HBsAg |  |  |  | 0.279 |
| Positive | 632 | 306(43.1%) | 326(56.9%) |  |
| Negative | 123 | 53 (43.1%) | 70 (56.9%) |  |
| AFP (ng/ml) |  |  |  | **0.001** |
| < 20 | 168 | 61(36.3%) | 107 (63.7%) |  |
| ≥ 20 | 587 | 298 (50.8%) | 289 (49.2%) |  |
| Cirrhosis |  |  |  | 0.137 |
| Yes | 616 | 285 (46.3%) | 331 (53.7%) |  |
| No | 139 | 74 (53.2%) | 65 (46.8%) |  |
| Tumor size (cm) |  |  |  | **<0.001** |
| < 5 | 190 | 63 (33.2%) | 127 (66.8%) |  |
| ≥ 5 | 565 | 296 (43.4%) | 269 (56.6%) |  |
| Tumor multiplicity |  |  |  | 0.845 |
| Single | 499 | 236 (47.4%) | 263 (52.6%) |  |
| Multiple | 256 | 123 (48.0%) | 133 (52.0%) |  |
| Differentiation |  |  |  | **<0.001** |
| Well-Moderate | 494 | 207 (41.9%) | 287 (58.1%) |  |
| Poor-undifferentiated | 261 | 152 (58.2%) | 109 (41.8%) |  |
| TNM |  |  |  | **<0.001** |
| I-II | 438 | 181 (41.3%) | 257 (58.7%) |  |
| III-IV | 317 | 178 (56.2%) | 139 (43.8%) |  |
| Vascular invasion |  |  |  | **<0.001** |
| Yes | 139 | 88(63.3%) | 51(36.7%) |  |
| No | 616 | 271 (44.0%) | 345 (56.0%) |  |
| Involucrum |  |  |  | **0.009** |
| Incomplete | 438 | 226 (51.6%) | 212 (48.4%) |  |
| Complete | 317 | 133 (42.0%) | 184 (58.0%) |  |
| LNM |  |  |  | 0.147 |
| No | 714 | 335 (46.9%) | 379 (53.1%) |  |
| Yes | 41 | 24 (58.5%) | 17 (41.5%) |  |

^a^Chi-square test; ^b^Median age; AFP, alpha-fetoprotein; HBV, hepatitis B virus; LNM, lymph node metastasis.

**Table S3. Univariate and multivariate analyses of clinicopathological and RDM1 expression for overall survival in overall cohort (n=755**).

| Variables | Univariate analysis | |  | | Multivariate analysis | | |
| --- | --- | --- | --- | --- | --- | --- | --- |
|  | HR (95% CI) | *P* value | |  | | HR (95% CI) | *P* value |
| **Overall survival** |  |  | |  | |  |  |
| Age (<49 vs. ≥49 years) | 0.891 (0.763-1.042) | 0.148 | |  | |  |  |
| Gender (female vs. male) | 0.843 (0.658-1.080) | 0.177 | |  | |  |  |
| HBV (positive vs. negative) | 1.256 (1.011-1.559) | 0.039 | |  | |  |  |
| Liver cirrhosis (yes vs. no) | 0.968 (0.789-1.189) | 0.759 | |  | |  |  |
| Tumor size (<5 vs. ≥5 cm) | 1.674 (1.390-2.016) | **<0.001** | |  | | 1.419 (1.171-1.719) | **<0.001** |
| Tumor multiplicity (single vs. multiple) | 1.719 (1.460-2.023) | **<0.001** | |  | | 1.263(1.043-1.531) | **0.017** |
| Invonucrum (absent vs. present) | 0.776 (0.662-0.911) | **0.002** | |  | | 0.903(0.763-1.067) | 0.231 |
| AFP (<20 vs. ≥20 ng/mL) | 1.284 (1.067-1.546) | **0.008** | |  | | 1.019 (0.840-1.235) | 0.852 |
| Vascular invasion (no vs. yes) | 2.649 (2.179 -3.221) | **<0.001** | |  | | 1.791 (1.446-2.218) | **<0.001** |
| Tumor differentiation | 1.541 (1.166-2.036) | **0.002** | |  | | 1.189 (0.889-1.589) | 0.243 |
| TNM (I vs. II-IV) | 1.909 (1.630-2.236) | **<0.001** | |  | | 1.537 (1.262-1.873) | **<0.001** |
| LNM (yes vs. no) | 1.774 (1.276-2.466) | **0.001** | |  | | 1.393 (1.995-1.952) | 0.054 |
| RDM1 expression (low vs. high) | 0.638 (0.546-0.746) | **<0.001** | |  | | 0.711 (0.606-0.836) | **<0.001** |

AFP, a-fetoprotein; HBsAg, hepatitis B surface antigen; HR, hazard ratio; CI, confidence interval.
